# Diffusion magnetic resonance imaging diagnostic relevance in pyogenic ventriculitis with an atypical presentation: a case report

**DOI:** 10.1186/1756-0500-7-149

**Published:** 2014-03-14

**Authors:** Lucio Marinelli, Carlo Trompetto, Leonardo Cocito

**Affiliations:** 1 Institute of Neurology, Department of Neuroscience, Rehabilitation, Ophthalmology, Genetics, Maternal and Child Health, University of Genova, Largo Daneo 3, 16132 Genova, Italy

**Keywords:** Pyogenic ventriculitis, Nosocomial infections, Staphylococcus Aureus, Neuroimaging, Diffusion-weighted magnetic resonance imaging, Corticosteroid theraphy

## Abstract

**Background:**

Pyogenic ventriculitis is a rare and severe cerebral infection characterized by the presence of suppurative fluid in the cerebral ventricles. It is a life-threatening condition and may present with an aspecific neurological picture. Brain imaging techniques usually demonstrate intraventricular debris and pus, but negative imaging along with a misleading clinical picture may delay the diagnosis.

**Case presentation:**

The described patient underwent a number of surgical procedures and eventually developed an unusual clinical picture characterized by psychomotor slowing, facial dyskinesias and myoclonic jerks without complaint of headache and in absence of meningeal irritation signs or focal neurological deficits. Cerebrospinal fluid cultural examination showed methicillin-resistant *Staphylococcus aureus* and vancomycin treatment lead to a complete recovery. Brain computed tomography scan was normal, while only diffusion magnetic resonance imaging sequences were able to define the presence of purulent material within the brain lateral ventriculi.

**Conclusion:**

The present case underlines the importance of taking into account the diagnosis of pyogenic ventriculitis even when the neurological picture does not match the suspect of a central nervous system infection. Moreover, brain computed tomography scan and standard magnetic resonance imaging sequences may be unable to confirm the diagnosis, whereas diffusion-weighted sequences prove a unique role in diagnosing cerebral pyogenic ventriculitis.

## Background

Pyogenic ventriculitis (also called ventricular empyema or pyocephalus) is a rare and severe cerebral infection characterized by the presence of suppurative fluid in the cerebral ventricles. The clinical picture is usually characterized by headache, vomiting, impaired consciousness with fever and meningeal signs [1]. Although a life threatening condition requiring the earliest diagnosis, symptoms and signs of pyogenic ventriculitis may sometimes be subtle and aspecific [2]. Among a large number of bacteria responsible for nosocomial infections, methicillin resistant *Staphylococcus aureus* (MRSA) was detected in many patients with pyogenic ventriculitis [2,3]. The possible mechanisms of infection of the ventricular system include hematogenous spread to the subependyma of the choroid plexus, diffusion by contiguity from a brain abscess or direct implantation secondary to trauma or surgical procedure [4]. Brain imaging techniques can demonstrate intraventricular debris and pus, representing the most common signs of ventriculitis [3-5]; other findings may include hydrocephalus and periventricular magnetic resonance abnormalities reflecting inflammatory changes [3].

We report a patient with pyogenic ventriculitis with an atypical neurological presentation, in whom the diffusion magnetic resonance imaging (MRI) findings were crucial for the diagnosis.

## Case presentation

**Figure 1:**
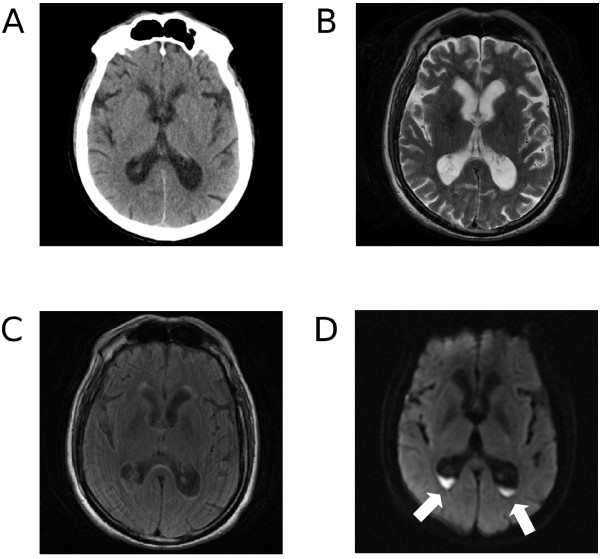
**Brain neuroimaging studies performed on the day when the diagnosis of ventriculitis was made.** Only the diffusion sequences clearly show the intraventricular debris. **A**. computed tomography scan; **B**. magnetic resonance T2 sequence; **C**. magnetic resonance FLAIR sequence; **D**. magnetic resonance diffusion sequence. Purulent material inside the ventriculi is indicated by the arrows.

A 66-year old man, who had been a heavy smoker for longer than 30 years, had a remote history of hypertension, hypothyroidism, and repeated surgical procedures (laparotomic surgery for appendicitis at 29, L5-S1 laminectomy at 44 and right hip surgery at 60). At the age of 65 he was diagnosed with a cancer in the sigmoid-rectum (pT3 pN1a adenocarcinoma) and underwent laparoscopic left hemicolectomy. Two days after the intervention, stump dehiscence required further laparotomic surgery. The clinical course was then complicated by bowel obstruction and sclerosing peritonitis which had to be surgically treated 20 and 32 days after the first surgery. During the latter procedure, the microbiological analysis of the surgical wound indicated the presence of a MRSA, and intravenous meropenem 3g/day was started. On day 35 since the first intervention, another surgery was needed because of the development of hemoperitoneum. The patient received repeated transfusions because of the worsening anemia. On day 48 he started developing a renal failure, which eventually required a kidney biopsy leading to the diagnosis of IgA nephropathy. The clinical conditions worsened during the following nine weeks, with fever up to 38.6°C and blood cultural examination positive for MRSA. He was treated with ceftriaxone, and then with methylprednisolon 40mg/day for 3 days. Up to this moment he had never had either neurological or psychiatric symptoms; some days later, however, he became anxious and restless and had a relapse of mild fever. He did not complain of headache and showed psychomotor retardation with preserved orientation and without any significant consciousness impairment. A neurologic consultation indicated an amimic face with bilateral myosis and reduced blinking. A preexisting and already known mild postural tremor was accompanied by almost continuous stereotyped limb movements, generalized weakness and suction-like mouth dyskinesias; no signs of meningeal irritation were found. A brain computed tomography (CT) scan disclosed no recent lesions (Figure 1A) and electroencephalogram (EEG) showed a diffuse slowing without evidence of epileptic activity. A brain MRI showed a mild enlargement of the lateral ventricles, which were surrounded by a slight increased signal in the FLAIR sequences (Figure 1B and C). A signal abnormality indicating dense material inside the ventricles was clearly recognizable in the diffusion sequences (Figure 1D), which strongly supported infection. Spinal tap was then performed and cerebrospinal fluid (CSF) showed increased leukocytes (1000/mm3, normal values 0-5) and protein content (8460 mg/l, normal values 150-450); glucose was not detectable and MRSA was found at the cultural examination. Vancomycin was started with an initial dosage of 1g/48h, later on raised to 1.5g/day because of low vancomycin plasma levels. Renal function did not worsen in the days during vancomycin administration. The patient was no longer febrile and showed a gradual improvement of clinical condition. Dexamethasone 8mg/day was administered as adjuvant therapy. Five days later, the stereotyped movements had disappeared, although the slight preexisting postural tremor was still superimposed by occasional myoclonic jerks and kinetic tremor in the left upper limb. Further two weeks later the neurological examination was negative, the leukocytes count in the CSF was decreased to 64/mm3, CSF glucose was still moderately low (27mg/dL, normal values 40-85), protein content was normal (294 mg/l) and both blood and CSF cultural examinations were negative. Vancomycin was replaced by linezolid to avoid further kidney damage, and the patient could be discharged to home after 7 weeks since the diagnosis of CNS infection.

## Conclusions

The reported patient had pyogenic ventriculitis due to infection by MRSA. The most important features of his clinical history were the background of malignancy and the repeated complications of surgery leading to an unusually high number of abdominal surgical procedures. It is conceivable that all this has delayed the healing process allowing the MRSA to infect the surgical wound and eventually disseminate, in a patient whose clinical status was impaired by nephropathy and other diseases. Moreover, an additional role may have been played by the corticosteroid therapy, which could on one side have facilitated the spreading of MRSA into the central nervous system by lowering the patient’s immunological defenses, on the other be responsible for the absence of signs of meningeal irritation and the peculiar clinical picture. The clinical manifestations of pyogenic ventriculitis are often aspecific, since consciousness impairment may hide other symptoms, and neurological focal deficits are hardly found when there are no abscesses or other lesions involving the brain. Our patient presented with a complex and atypical neurological picture, mainly characterized by motor symptoms and behavioral abnormalities, but with no evidence of meningeal irritation, headache or focal deficits (considering “focal” those neurological signs of symptoms suggesting a lesion of a specific structure within the neurological system). Neuroimaging had a critical role in the diagnosis. CT scan was quite unremarkable and only the diffusion MRI sequences could clearly indicate the presence of purulent material within the ventricles. Recent studies have emphasized the importance of diffusion sequences in the diagnosis of pyogenic ventriculitis [2-4]. Indeed, the relatively high viscosity, hypercellularity and binding of water to macromolecules have been suggested as explanations for the restricted water diffusion observed in the purulent material [2]. Conversely, FLAIR sequences are considered useful in detecting periventricular high signal and ependymal enhancement [5]. Mild ventricular enlargement and increased periventricular signal were also detected in our patient, but specificity of these findings was obviously low.

In conclusion our patient underscores the unique role of MR diffusion sequences to detect purulent material inside the ventricles, thus warranting the diagnosis of pyogenic ventriculitis; this is even more true when the pathological process is limited. Moreover, the clinical features of this case point out that the diagnosis of pyogenic ventriculitis should be considered also when signs of meningeal irritation are poor or even absent. In fact, the clinical evidence pointing to an acute infection of the brain may be lacking, and a misleading neurological picture with mainly motor and behavioral manifestations may occur. Finally, it should be underlined that ventricular infection may follow an hematogenic route, in absence of a brain abscess, trauma or a surgical procedure.

## Consent

Written informed consent was obtained from the patient for publication of this Case Report and any accompanying images. A copy of the written consent is available for review by the Editor-in-Chief of this journal.

## Competing interests

The authors declare that they have no competing interests.

## Authors’ contributions

LM: patient examination, data collection, writing the first draft of the manuscript; CT: manuscript revision and discussion; LC: patient examination, manuscript revision and discussion. All authors read and approved the final manuscript.
